# Human Clade 2.3.4.4 A/H5N6 Influenza Virus Lacks Mammalian Adaptation Markers and Does Not Transmit via the Airborne Route between Ferrets

**DOI:** 10.1128/mSphere.00405-17

**Published:** 2018-01-03

**Authors:** Sander Herfst, Chris K. P. Mok, Judith M. A. van den Brand, Stefan van der Vliet, Miruna E. Rosu, Monique I. Spronken, Zifeng Yang, Dennis de Meulder, Pascal Lexmond, Theo M. Bestebroer, J. S. Malik Peiris, Ron A. M. Fouchier, Mathilde Richard

**Affiliations:** a Department of Viroscience, Postgraduate School Molecular Medicine, Erasmus MC, Rotterdam, The Netherlands; b HKU-Pasteur Research Pole, School of Public Health, HKU Li Ka Shing Faculty of Medicine, The University of Hong Kong, Hong Kong, SAR, People’s Republic of China; c State Key Laboratory of Respiratory Disease, National Clinical Research Center for Respiratory Disease, First Affiliated Hospital of Guangzhou Medical University, Guangzhou, People’s Republic of China; d Macau University of Science and Technology, Macau, People’s Republic of China; Boston University School of Medicine

**Keywords:** 2.3.4.4, ferrets, highly pathogenic avian influenza, pathogenesis, transmission

## Abstract

Since their emergence in 1997, A/H5N1 influenza viruses of the A/goose/Guangdong/1/96 lineage have diversified in multiple genetic and antigenic clades upon continued circulation in poultry in several countries in Eurasia and Africa. Since 2009, reassortant viruses carrying clade 2.3.4.4 hemagglutinin (HA) and internal and neuraminidase (NA) genes of influenza A viruses of different avian origin have been detected, yielding various HA-NA combinations, such as A/H5N1, A/H5N2, A/H5N3, A/H5N5, A/H5N6, and A/H5N8. Previous studies reported on the low pathogenicity and lack of airborne transmission of A/H5N2 and A/H5N8 viruses in the ferret model. However, although A/H5N6 viruses are the only clade 2.3.4.4 viruses that crossed the species barrier and infected humans, the risk they pose for human health remains poorly characterized. Here, the characterization of A/H5N6 A/Guangzhou/39715/2014 virus *in vitro* and in ferrets is described. This A/H5N6 virus possessed high polymerase activity, mediated by the E627K substitution in the PB2 protein, which corresponds to only one biological trait out of the three that were previously shown to confer airborne transmissibility to A/H5N1 viruses between ferrets. This might explain its lack of airborne transmission between ferrets. After intranasal inoculation, A/H5N6 virus replicated to high titers in the respiratory tracts of ferrets and was excreted for at least 6 days. Moreover, A/H5N6 virus caused severe pneumonia in ferrets upon intratracheal inoculation. Thus, A/H5N6 virus causes a more severe disease in ferrets than previously investigated clade 2.3.4.4 viruses, but our results demonstrate that the risk from airborne spread is currently low.

**IMPORTANCE** Avian influenza A viruses are a threat to human health, as they cross the species barrier and infect humans occasionally, often with severe outcome. The antigenic and genetic diversity of A/H5 viruses from the A/goose/Guangdong/1/96 lineage is increasing, due to continued circulation and reassortment in poultry, posing a constant risk for public health and requiring regular risk assessments. Here we performed an in-depth characterization of the properties of the newly emerged zoonotic A/H5N6 virus *in vitro* and in ferrets. The lack of airborne transmission in the ferret model indicates that A/H5N6 virus does not pose a direct public health threat, despite the fact that it can replicate to high titers throughout the respiratory tracts of ferrets and cause more severe disease than other clade 2.3.4.4 viruses.

## Abstract (precis)

Avian influenza A viruses are a threat to human health, as they cross the species barrier and infect humans occasionally, often with severe outcome. The antigenic and genetic diversity of A/H5 viruses from the A/goose/Guangdong/1/96 lineage is increasing, due to continued circulation and reassortment in poultry, posing a constant risk for public health and requiring regular risk assessments. Here we performed an in-depth characterization of the properties of the newly emerged zoonotic A/H5N6 virus *in vitro* and in ferrets. The lack of airborne transmission in the ferret model indicates that A/H5N6 virus does not pose a direct public health threat, despite the fact that it can replicate to high titers throughout the respiratory tracts of ferrets and cause more severe disease than other clade 2.3.4.4 viruses.

## INTRODUCTION

Highly pathogenic avian influenza (HPAI) A/H5 viruses of the A/goose/Guangdong/1/1996 (GsGd) lineage first emerged in Hong Kong in 1997 (1). Since 2003, these viruses have spread to Eurasia and Africa, and the GsGd lineage is the only lineage of HPAI viruses that are endemic in poultry. As a result of continuous circulation in poultry, the hemagglutinin (HA), the main antigenic determinant of influenza A viruses, of A/H5 GsGd viruses diversified in several genetic and antigenic clades (2). Moreover, due to the segmented nature of the influenza A virus genome, viruses carrying GsGd HAs reassorted with cocirculating low-pathogenic avian influenza (LPAI) viruses from domestic and wild birds, increasing the genetic diversity of A/H5 GsGd viruses. In particular, the H5 HA of clade 2.3.4.4 was found to reassort frequently with neuraminidase (NA) genes of other subtypes than N1, yielding A/H5 viruses with various HA-NA combinations: A/H5N1, A/H5N2, A/H5N3, A/H5N5, A/H5N6, and A/H5N8 (3).

In contrast to A/H5N8 viruses, which spread worldwide in a global fashion via wild migratory birds (4), the distribution of A/H5N6 viruses has until recently been limited to China, Laos, and Vietnam. In China, they have replaced A/H5N1 viruses and became the dominant A/H5 GsGd circulating virus in poultry (5). However, A/H5N6 viruses genetically related to the viruses from China were isolated during the 2016-2017 winter from wild migratory birds in Korea and Japan, where they also caused poultry outbreaks (6–8), suggesting a potential for A/H5N6 viruses to spread via wild bird migration also.

A/H5N6 viruses are the only clade 2.3.4.4 viruses that have crossed the species barrier and infected humans. The first human cases of infection with the two circulating lineages of A/H5N6 viruses were both reported in China (9, 10). As of 8 December 2017, a total of 17 patients infected with A/H5N6 have been reported, and 6 of these patients died (11). Almost all patients infected with A/H5N6 viruses had documented exposure to infected poultry at live poultry markets, suggesting poultry exposure as a potential source of zoonotic transmission (12). The first reassortant A/H5N6 viruses identified in April 2013 and the viruses isolated from the first three human cases in 2014 were carrying the internal genes of A/H5N1 viruses. Based on the origin of their HA and NA genes, these viruses belong to two genotypes (G1 and G2). After 2014, multiple other genotypes emerged upon reassortment with circulating A/H9N2 or A/H7N9 viruses (5, 12, 13), resulting in the definition of 34 genotypes (5).

The global spread via wild birds and complex reassortment history of clade 2.3.4.4 viruses required a detailed molecular and phenotypic characterization, especially of the newly emerged A/H5N6 viruses. Several research groups previously reported on the low pathogenicity and lack of airborne transmissibility in the ferret model of the Korean, European, and North American A/H5N8 viruses and North American A/H5N2 viruses, probably partially due to the presence of internal genes originating from wild bird LPAI viruses (14–16). In contrast, A/H5N6 viruses carry internal genes coming from A/H5N1, A/H7N9, and A/H9N2 viruses, which are poultry-adapted viruses that have themselves caused zoonotic infections or acted as donors of internal genes to most other zoonotic influenza viruses (5).

Here, we focused our investigation on A/H5N6 A/Guangzhou/39715/2014 (GZ/14) virus, which belongs to genotype G1 and caused one of the first human cases of A/H5N6 infection in 2014 (17). *In vitro* characterization of phenotypes that have been associated with airborne (respiratory droplets and aerosols) transmission of A/H5N1 viruses, such as receptor binding preference, thermostability, pH stability, and polymerase activity was performed. The potential of A/H5N6 GZ/14 to transmit via the airborne route between ferrets was assessed, as well as its pathogenicity in the ferret model. While our study suggests that the public health risk posed by this A/H5N6 virus is low, the A/H5N6 GZ/14 showed a high polymerase activity mediated by the E627K substitution in PB2, replicated to higher titers in the respiratory tracts of ferrets and was more pathogenic than a clade 2.3.4.4 A/H5N8 virus (14).

## RESULTS

### A/H5N6 GZ/14 virus binds to avian-type receptors.

The influenza A virus HA protein binds to host cells via sialylated receptors. Avian and human viruses bind α2,3-linked sialic acids (α2,3-SA, avian-type receptors) and α2,6-linked sialic acids (α2,6-SA, human-type receptors), respectively. In order to assess the receptor specificity of A/H5N6 GZ/14, a resialylated red blood cell assay was performed. Briefly, SA were stripped off turkey red blood cells (TRBCs) upon sialidase treatment (*Vibrio cholerae* neuraminidase [VCNA]), and either α2,3-SA or α2,6-SA were subsequently rebuilt using specific sialyltransferases. Both control viruses, the avian A/H5N1 virus A/Indonesia/5/2005 (IN/05) and the human A/H3N2 virus A/Netherlands/213/2003 (NL/03), displayed the expected receptor attachment pattern by binding exclusively to α2,3-SA and α2,6-SA, respectively (Table 1). Viruses of clade 2.3.4.4, A/H5N6 GZ/14 and A/H5N8 A/chicken/Netherlands/EMC-3/2014 (ck/NL/14), displayed a phenotype of typical avian viruses by binding exclusively to α2,3-SA.

**TABLE 1:** Receptor specificity of the different viruses as determined by a modified TRBC hemagglutination assay^a^

Virus	HA titer with the following erythrocytes (TRBCs) (HAU^b^/25 µl)			
	Untreated	VCNA	α2,3^c^	α2,6^c^
A/H5N1 IN/05 (α2,3-SA control)	64	<1	64	<1
A/H3N2 NL/03 (α2,6-SA control)	32	<1	<1	32
A/H5N6 GZ/14	64	<1	64	<1
A/H5N8 ck/NL/14	64	<1	64	<1

a Two independent experiments were performed, and results presented here are from one representative experiment. VCNA, *Vibrio cholerae* neuraminidase.

b HAU, hemagglutination units.

c SA stripped off TRBCs upon sialidase treatment, and either α2,3-SA or α2,6-SA was subsequently rebuilt using specific sialyltransferases.

### A/H5N6 GZ/14 HA is unstable.

**FIG 1:**
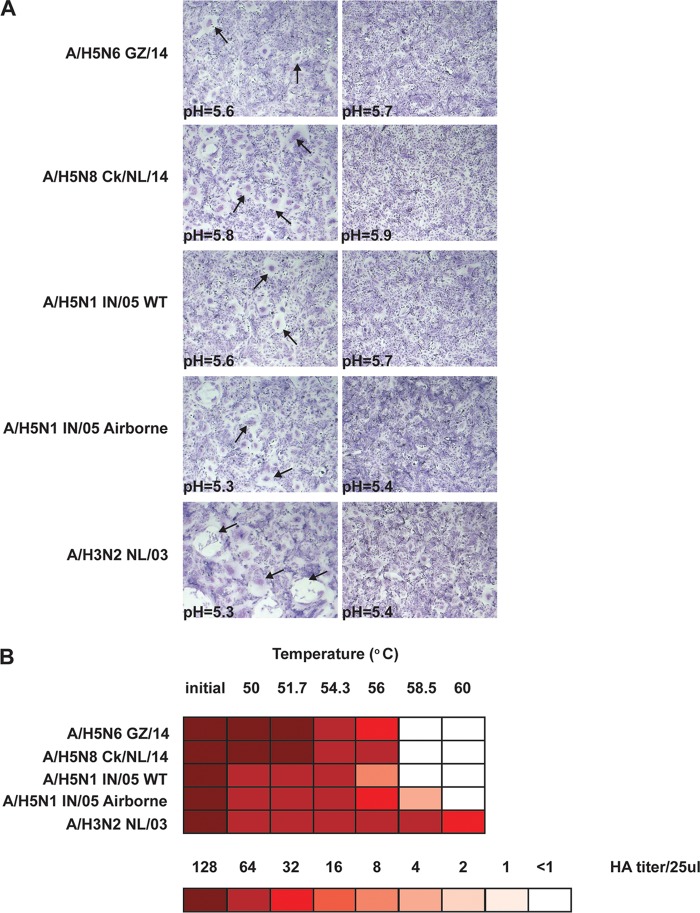
pH threshold of fusion stability and thermostability of clade 2.3.4.4 A/H5 viruses. (A) Syncytium formation in Vero-118 cells upon expression of HAs from different A/H5 viruses and acidification at different pH values. Pictures corresponding to the last pH at which maximum syncytium formation was observed (defined as the pH threshold of fusion) and 0.1 pH unit higher are shown. Some areas showing fusion (syncytia) are indicated by black arrows. Three independent experiments were performed, and results presented here are from one representative experiment. (B) HA protein thermostability was measured by the ability of viruses to agglutinate TRBCs after incubation for 30 min at the indicated temperatures (Celsius). Colors indicate the HA titers upon treatment at various temperatures as shown in the color key (HA titer/25 μl). Three independent experiments were performed, and results presented here are from one representative experiment.

Upon virus attachment to SA receptors on the cell surface and internalization into endosomes, a low-pH-triggered conformational change of HA mediates fusion of the viral and endosomal membranes to release the viral genome in the cytoplasm (18). In order to assess the acid stability of the HA of A/H5N6 GZ/14, a syncytium formation assay was performed to measure the pH threshold required for HA-mediated cell-to-cell fusion. Vero cells were transfected with plasmids expressing different HAs and exposed to trypsin to cleave and activate the HA, followed by acidification of the cell culture by a pH gradient ranging from pH 4.8 to 5.9. Visual inspection of the cell cultures for the presence of syncytia (multinucleated cells) was used to determine the pH threshold triggering the conformational change and subsequent membrane fusion (Fig. 1A). Control HAs, the A/H5N1 IN/05 wild-type (WT) HA (unstable), the A/H5N1 IN/05 HA carrying airborne-transmission substitutions (H103Y, T156A, Q222L, and G224S [H5 numbering used throughout]) (stable), and the human A/H3N2 NL/03 HA (stable), were included. The pH values at which cell-to-cell fusion was triggered by A/H5N6 GZ/14 HA and A/H5N8 ck/NL/14 HA were 5.6 and 5.8, respectively, which were similar to or higher than those of the unstable A/H5N1 IN/05 WT HA (pH 5.6) and higher than the two stable HAs, A/H5N1 IN/05 airborne (pH 5.3) and A/H3N2 NL/03 (pH 5.3).

The conformational change of HA from a metastable nonfusogenic state to a stable fusogenic state can also be triggered at neutral pH when the HA is exposed to increasing temperatures (19). Therefore, the thermostability was assessed using a temperature sensitivity assay to further assess HA stability (Fig. 1B). Viruses were exposed to a temperature gradient ranging from 50 to 60°C for 30 min, and the HA titer was subsequently recorded. In agreement with the results of the fusion assay, A/H5N6 GZ/14 and A/H5N8 ck/NL/14 viruses were less stable than the human A/H3N2 and the airborne transmissible A/H5N1 virus.

### A/H5N6 GZ/14 possesses a high polymerase activity, mediated by the E627K substitution in PB2.

**FIG 2:**
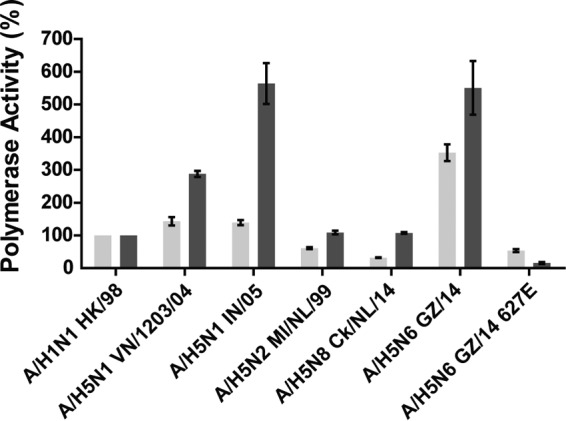
Polymerase activity of clade 2.3.4.4 A/H5 viruses. 293T cells were transfected with plasmids containing PB2, PB1, PA, and NP genes, a luciferase (firefly) reporter plasmid, and a control luciferase (*Renilla*) reporter plasmid. After transfection, the 293T cells were cultured at 33°C (light gray) and 37°C (dark gray) for 24 h, and luciferase activity was evaluated from the cell extracts. Results are presented as the means ± standard deviations (error bars) of polymerase activity of virus compared to the polymerase activity of the human A/H1N1 virus, HK/98, which was set at 100%, and are averages from three independent experiments.

To measure the polymerase activity of A/H5N6 GZ/14, minireplicon assays were performed by transfecting 293T cells with plasmids expressing the polymerase genes (PB2, PB1, and PA) and the nucleoprotein (NP) of different GsGd viruses, human A/H1N1 virus or avian A/H5N2 virus, luciferase (*Renilla*) as an internal control, and luciferase (firefly) as a reported gene. The experiment was performed at 33°C and 37°C. Polymerase activities were expressed as a percentage of the polymerase activity of the human A/H1N1 virus, HK/98, which was set at 100% for both temperatures. At 33°C, the polymerase activity of the A/H5N6 GZ/14 was higher than that of other viruses tested, including A/H5N1 IN/05 and A/H5N8 ck/NL/14 (Fig. 2). At 37°C, the polymerase activity of A/H5N6 GZ/14 was similar to that of A/H5N1 IN/05 and higher than that of the other viruses tested. A/H5N6 GZ/14 possesses a lysine at position 627 in the polymerase basic 2 (PB2), which has been associated with adaptation of avian viruses to mammalian hosts (20–22). In order to understand whether the high polymerase activity of A/H5N6 GZ/14 was the result of the presence of PB2-627K, the PB2 gene of A/H5N6 GZ/14 was mutated to the avian genotype (K627E). The polymerase activities of the A/H5N6 GZ/14 PB2-627E were 7 and 32 times lower than those of A/H5N6 GZ/14 at 33°C and 37°C, respectively. It was also lower than that of A/H5N1 viruses isolated from human cases of infection (A/H5N1 IN/05 and A/H5N1 VN/1203/04) and comparable to that of other avian viruses, A/H5N2 Ml/NL/99 and A/H5N8 ck/NL/14. These observations demonstrated that the high polymerase activity of A/H5N6 GZ/14 was mediated by the E627K substitution in PB2.

### A/H5N6 GZ/14 does not transmit between ferrets via the airborne route.

**FIG 3:**
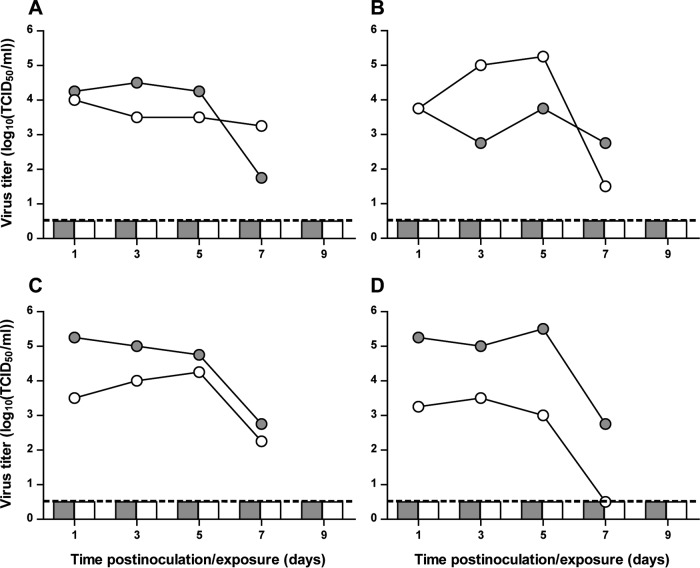
Lack of airborne transmission of A/H5N6 GZ/14 between ferrets. Data for individual transmission experiments are shown in each panel, with virus shedding from donor and recipient ferrets shown as lines and bars, respectively. Gray circles and squares represent shedding from the throat; white circles and squares represent shedding from the nose. All virus titers were determined by endpoint titration in MDCK cells. The lower limit of detection is indicated by the dashed lines.

Human influenza viruses are known to be transmitted from person to person via the airborne route by aerosols or respiratory droplets. Therefore, it is of importance to investigate the airborne transmissibility of emerging influenza A viruses. Here, airborne transmission experiments were performed in ferrets. Briefly, one day after donor ferrets were inoculated with A/H5N6 GZ/14, naive recipient ferrets were placed in an adjacent cage, separated from the donor cage by a steel grid, allowing viruses to be transmitted only via the airborne route. Virus shedding from the inoculated ferrets was high, with titers up to 10^5.25^ 50% tissue culture infective doses (TCID_50_)/ml, and long-term, until 7 days postinoculation (dpi) (Fig. 3). Upon exposure, no replicating virus was detected in any of the recipient ferrets. Lack of transmission was confirmed by the absence of seroconversion 14 days postexposure in all recipient ferrets (data not shown).

### **A/H5N6 GZ/14 is more pathogenic than a clade** 2.3.4.4 **A/H5N8 virus.**

In order to assess the pathogenicity of A/H5N6 GZ/14, six ferrets were inoculated intranasally with 10^6^ TCID_50_ of virus. Daily, the animals were monitored for body weight and clinical signs. Nose and throat swabs were also collected daily. At 3 and 6 dpi, three animals were sacrificed, and necropsies were performed. Data from this experiment were compared to historical data on intranasal challenge of ferrets with A/H5N8 ck/NL/14 and A/H5N1 IN/05 (14).

**FIG 4:**
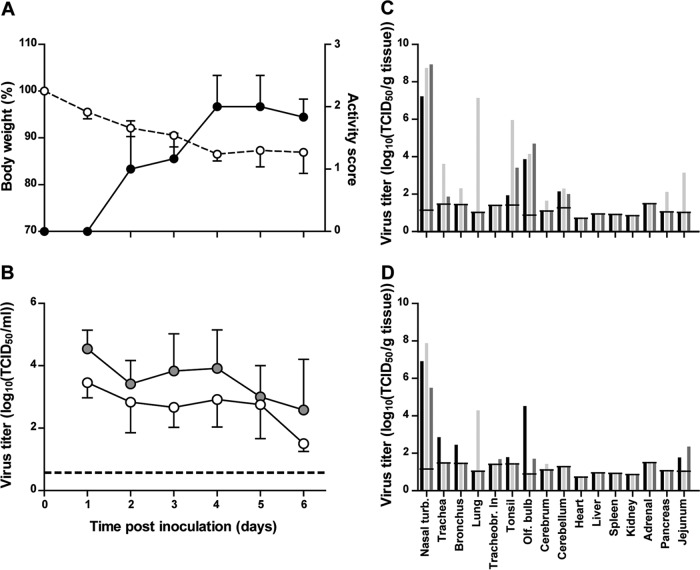
Body weight, activity status, and virus titers in swabs and tissues of ferrets upon intranasal inoculation with A/H5N6 GZ/14. (A) Average body weight is represented by the dashed line and the open white circles (left *y* axis). Average activity status is represented by the solid line and the closed black circles (right *y* axis). Activity status was scored daily as follows: 0, alert and playful; 1, alert and playful only when stimulated; 2, alert but not playful when stimulated; 3, neither alert nor playful when stimulated. (B) Average virus titers in the throat and nasal swabs are shown by the gray circles and white circles, respectively. (C) Virus titers in tissues collected at 3 dpi are shown for individual ferrets in black, light gray, and dark gray. (D) Virus titers in tissues collected at 6 dpi are shown for individual ferrets in black, light gray, and dark gray. All virus titers were determined by endpoint titration in MDCK cells. In panel B, the lower limit of detection is indicated by the dotted line. In panels C and D, the lower limit of detection is indicated by the short horizontal lines spanning the three bars for each tissue. All error bars represent standard deviations. Nasal turb., nasal turbinates; Tracheobr. ln, tracheobronchial lymph node; Olf. bulb, olfactory bulb.

A/H5N6 GZ/14-inoculated animals lost on average 13% of their starting body weight (ranging from 9 to 18% at 6 dpi) (Fig. 4A). This body weight loss was intermediate between that of A/H5N8-inoculated animals (3%) and that of A/H5N1-inoculated animals (21%). At 3 dpi, all animals were less active and exhibited ruffled fur and watery eyes. One ferret was particularly weak, especially in the hind legs and did not move when stimulated. At 4 dpi, all ferrets presented with ruffled fur and with weak hind legs, showed inappetence, and kept their eyes slightly closed. At 6 dpi, all animals regained activities, and their body weight stabilized. To summarize, A/H5N6 GZ/14-inoculated animals showed more severe clinical signs than A/H5N8-inoculated animals, such as inactivity or ruffled fur, as reflected by the global clinical score (Fig. 4A). However, they did not progress to visible signs of pneumonia as observed in A/H5N1-inoculated animals (14).

The shedding of A/H5N6 GZ/14 from the respiratory tracts of ferrets, as measured by virus titration of throat and nose swab samples, was high and prolonged, similar to that of A/H5N1 IN/05, and longer and higher in titers than those of A/H5N8 ck/NL/14 (Fig. 4B).

A/H5N6 GZ/14 virus was detected by virus titration at high titers in the nasal turbinates, both at 3 dpi and 6 dpi (mean titers of 10^8.3^ and 10^6.8^ TCID_50_/g of tissue, respectively) (Fig. 4C and D). Moreover, A/H5N6 GZ/14 virus was detected at 3 dpi in more tissues of the respiratory tract, such as the lung and tonsils, and at higher titers than of A/H5N8 ck/NL/14. At 3 dpi, A/H5N6 GZ/14 was indeed detected at a high titer (10^7.1^ TCID_50_/g of tissue) in the lung of one animal. A/H5N6 GZ/14 was also detected at 3 dpi in extrarespiratory tissues such as the olfactory bulb (mean titers of 10^4.2^ TCID_50_/g of tissue). At 6 dpi, A/H5N6 GZ/14 was detected only in the lung of one animal, which was the animal that had lost the most weight (18%) (Fig. 4D). The low virus titers in the brain, pancreas, and intestine at 3 dpi and 6 dpi were not confirmed by immunohistochemical analysis (IHC) detecting with the viral NP (Fig. 4C and D; Table 2).

**TABLE 2:** Virus antigen expression in tissues of ferrets inoculated intranasally or intratracheally with A/H5N6 GZ/14

System and tissue	Virus antigen expression in ferrets^a^								
	Intranasal challenge						Intratracheal challenge		
	Necropsied at 3 dpi			Necropsied at 6 dpi			F1	F2	F3
	F1	F2	F3	F4	F5	F6			
Respiratory									
Nasal respiratory epithelium (rostral)	−	−	−	−	−	−	−	−	−
Nasal respiratory epithelium (caudal)	−	+	+	+/−	+	+/−	−	−	−
Nasal olfactory epithelium	++	+++	+++	+	++	+	−	−	−
Nasal glands	+	+	+/−	+	+/−	+/−	−	−	−
Nasal stratified epithelium	−	−	−	−	−	−	−	−	−
Larynx	−	+/−^b^	−	−	−	+/−	−	−	−
Trachea	−	−	−	−	+/−	−	−	−	−
Tracheal glands	−	−	−	−	−	−	−	−	−
Bronchi	−	−	−	−	−	−	−	−	−
Bronchial glands	−	−	−	−	−	−	−	−	−
Bronchioles	−	+/−	−	−	−	−	−	−	−
Alveoli, pneumocyte type 1	−	+/−	−	−	−	−	+/−	++	++
Alveoli, pneumocyte type 2	−	++	−	−	−	−	+	++	++
Alveolar macrophages	−	+	−	−	−	−	−	−	−
Tonsil	−	−	X	X	X	−	X	−	−
Tracheobronchial lymph node	−	+/−	X	−	−	−	+/−	+/−	−

Extrarespiratory									
Heart	−	−	−	−	−	−	−	−	−
Liver	−	−	−	−	−	+/−^c^	−	−	−
Spleen	−	−	−	−	−	−	−	−	−
Kidney	−	−	−	−	−	−	−	−	−
Adrenal	−	−	−	−	−	−	−	−	−
Esophagus	−	−	−	−	−	−	−	−	−
Duodenum	−	−	−	−	−	−	−	−	−
Jejunum	−	−	−	−	−	−	−	−	−
Colon	−	−	−	−	−	−	−	−	−
Pancreas	−	−	−	−	−	+/−^d^	−	−	−
Mesenteric lymph node	−	X	−	−	−	−	−	−	−

Nervous									
Nasal nerves	−	−	−	−	−	−	−	−	−
Trigeminus nerve	−	−	−	−	−	−	−	−	−
Olfactory bulb	+/−	+/−	−	−	+/−	+/−	−	−	−
Cerebrum	−	−	−	−	−	−	−	−	−
Cerebellum	−	−	−	−	−	−	−	−	−
Eye lid	−	−	−	−	−	−	−	−	−

a Viral antigen expression in individual ferrets (three ferrets per group). Ferrets are indicated as follows: F1, ferret 1; F2, ferret 2, etc. Symbols: −, no virus antigen (nucleoprotein) present; +/−, virus antigen expression in occasional cells; +, virus antigen expression in a few cells; ++, virus antigen expression in a moderate number of cells; +++, virus antigen expression in many cells; X, tissue sample not available.

b Virus antigen expression in occasional epithelial cells associated with mild mixed inflammatory infiltrate.

c Focal virus antigen expression in occasional cholangioductular epithelial cells associated with infiltration of a moderate number of macrophages and lymphocytes and of a few neutrophils and plasma cells.

d Virus antigen expression in occasional pancreatic ductular epithelial cells. Multifocally, there was moderate interstitial edema and infiltration of macrophages, neutrophils, lymphocytes, and plasma cells.

**FIG 5:**
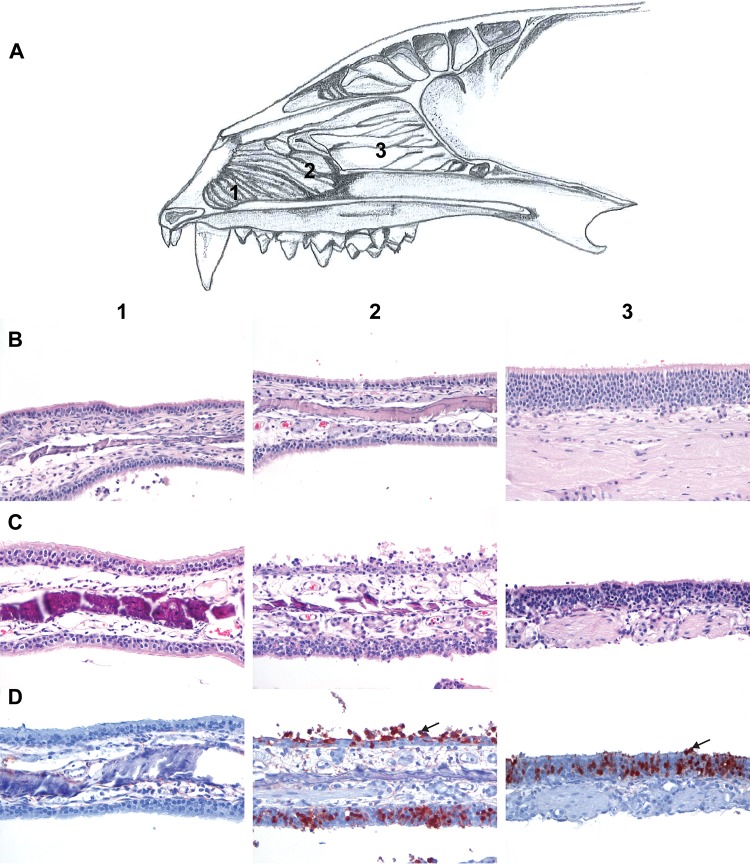
Histopathological and immunohistochemical analyses of nasal epithelium samples from ferrets inoculated intranasally with A/H5N6 GZ/14, collected at 3 dpi. (A) Schematic drawing of a cross section of a ferret head illustrating the anatomical sites of the rostral respiratory epithelium (1), caudal respiratory epithelium (2), and olfactory epithelium (3). (B) Hematoxylin and eosin (HE) staining of nasal turbinates of a naive ferret showing the different epithelia. (C) HE staining of nasal turbinates of a ferret inoculated with A/H5N6 GZ/16 showing the different epithelia. (D) Influenza A virus NP antigen expression as determined by IHC in the different epithelia of the nasal turbinates of a ferret inoculated with A/H5N6 GZ/16. Arrows indicate cells positive for virus antigen NP expression. The pictures in panels C and D were taken from the nasal turbinates of one animal inoculated with A/H5N6 GZ/16 and serve as representative images for all animals. Magnification, ×200.

At 3 dpi, high titers in the nasal turbinates correlated with the presence of virus antigen in the caudal respiratory epithelium and more abundantly in the olfactory epithelium in the nose of all ferrets (Fig. 5; Table 2). Virus antigen expression was associated with moderate epithelial necrosis and infiltration of some neutrophils and macrophages in the caudal respiratory epithelium and with moderate to severe necrosis and inflammation in the olfactory epithelium. Gross pathology revealed multifocal moderate gray-red consolidation and multifocal mild emphysema in the lung of one ferret, in which virus titers were also detected (see above), with 50% of the lungs affected and a relative lung weight (RLW) of 0.9%, expressed as a percentage of the total body weight (Table 3). The RLW is a measure of the level of pulmonary inflammatory infiltrate and edema. In comparison, the median RLW of noninfected ferrets is 0.6% of total body weight, with a range from 0.5% to 0.8% (23). In the lungs of this ferret, virus antigen expression was observed in few pneumocytes, more of the type II than of the type I, and in a few alveolar macrophages (Table 2). Virus antigen expression was associated with a mild to moderate infiltration of neutrophils, alveolar macrophages, edema, and epithelial necrosis. Moreover, there was a multifocal peribronchiolar cuffing with lymphocytes, macrophages, and plasma cells. The other two ferrets presented with only 0% and 5% of the lung tissue affected (RLWs of 0.4% and 0.6%, respectively), and no virus antigen expression nor microscopic lesions were observed by histopathology (Tables 2 and 3). In the olfactory bulbs of two ferrets, virus antigen expression was observed in some cells that were associated with mild gliosis. In other tissues, antigen expression was not demonstrated, and there were no significant lesions (Table 2).

**TABLE 3:** Gross pathology of lungs of ferrets inoculated intranasally or intratracheally with A/H5N6 GZ/14

Parameter	Value (%) for parameter in ferrets^a^								
	Intranasal challenge						Intratracheal challenge		
	Necropsied at 3 dpi			Necropsied at 6 dpi			F1	F2	F3
	F1	F2	F3	F4	F5	F6			
Affected lung tissue	0	50	5	0	0	10	100	90	100
Relative lung wt^b^	0.4	0.9	0.6	0.8	0.7	0.7	1.6	1.3	1.6

a Ferrets are indicated as follows: F1, ferret 1; F2, ferret 2, etc.

b The relative lung weight was expressed as a percentage of the total body weight at the time of the necropsy. The median relative lung of noninoculated female ferrets is 0.6 (range, 0.5 to 0.8).

At 6 dpi, virus antigen distribution in the nasal epithelium (respiratory and olfactory) was similar to that of animals euthanized at 3 dpi albeit with fewer positive cells (Table 2). Virus antigen expression was also associated with inflammation but with less necrosis compared to 3 dpi. Moreover, one ferret presented lesions in 10% of his lungs characterized by mild consolidation (RLW of 0.7%), while the other two ferrets showed no lesions (RLWs of 0.7% and 0.8%). Microscopically, no virus antigen or lesions were observed in the lungs of animals euthanized at 6 dpi. In the olfactory bulbs of two ferrets, few cells in the glomerular zone were virus antigen positive associated with mild gliosis.

### A/H5N6 GZ/14 causes severe pneumonia upon intratracheal inoculation of ferrets.

Most humans infected with an HPAI A/H5 GsGd virus developed severe pneumonia resulting in acute respiratory distress syndrome. Differences in virus tropism and pathogenicity after different routes of inoculation of ferrets have already been reported (24), and it has been shown that intranasal inoculation does not necessarily result in the development of pneumonia, as seen in most human cases of infection. Therefore, in order to study the ability of A/H5N6 GZ/14 to cause pneumonia, three ferrets were inoculated intratracheally with 10^6^ TCID_50_ of virus. At 1 dpi, all animals were alert, but they were playful only after stimulation (activity status [AS] of 1). At 2 dpi, in the morning, one ferret was found dead, while the two other ferrets were less active than the day before (AS of 2). Later in the afternoon, these two ferrets were neither alert nor playful when stimulated (AS of 3). One animal presented with severe dyspnea and succumbed to the infection shortly after. The other ferret was very weak, demonstrated swollen conjunctiva and mild dyspnea and was euthanized because of its poor condition. The animal that died at 1 dpi had lost 6% of its starting body weight, and the other two ferrets had lost 12% and 10% of their starting body weights.

**FIG 6:**
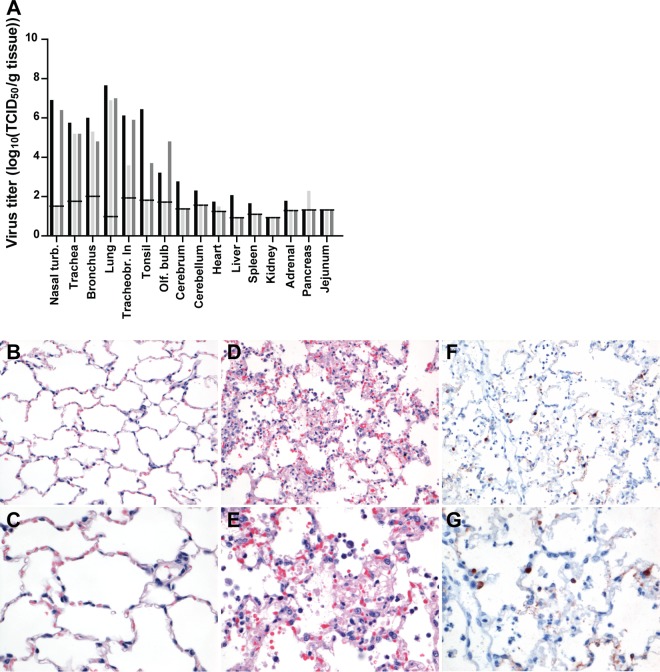
Virus titers in tissues of ferrets inoculated intratracheally with A/H5N6 GZ/14 and histopathological and immunohistochemical analyses of the lungs at the time of death. (A) Individual virus titers in tissues at the time of death/euthanasia. All titers were determined by endpoint titration in MDCK cells. The lower limit of detection is indicated by the short horizontal lines spanning the three bars for each tissue type. Nasal turb., nasal turbinates; Tracheobr. ln, tracheobronchial lymph node; Olf. bulb, olfactory bulb. (B) HE staining of a lung from a naive ferret. Magnification, ×200. (C) HE staining of a lung from a naive ferret. Magnification, ×400. (D) HE staining of a lung from an A/H5N6 GZ/14-inoculated ferret. Magnification, ×200. (E) HE staining of a lung from an A/H5N6 GZ/14-inoculated ferret. Magnification, ×400. (F) NP-positive staining of a lung from an A/H5N6 GZ/14-inoculated ferret. Magnification, ×200. (G) NP-positive staining of a lung from an A/H5N6 GZ/14-inoculated ferret. Magnification, ×400. The pictures shown in panels D and E and panels F and G show the lung from one animal and serve as representative images for all animals.

At the time of death or euthanasia, between 1 dpi and 2 dpi depending on the animal, virus titers were very high throughout the respiratory tract and with the highest titers in the lungs (mean titers of 10^7.2^ TCID_50_/g of tissue) (Fig. 6A).

Gross pathological analysis revealed a severe diffuse to coalescing pulmonary consolidation affecting 90 to 100% of the lungs of all animals, characterized by dark red and depressed lung parenchyma. The RLWs of these ferrets were between 1.3% and 1.6% (Table 3). The ferrets had enlarged tracheobronchial lymph nodes, approximately two to three times the normal size. Microscopically, there was multifocal coalescing to diffuse expression of virus antigen in many type II pneumocytes, in a moderate number of type I pneumocytes, and few macrophages. Virus antigen expression was associated with multifocal coalescing to severe diffuse alveolar damage (DAD) (Fig. 6D to G). DAD was characterized by severe epithelial necrosis, large amounts of intraluminal proteinaceous exudate (edema), fibrin, extravasated erythrocytes (hemorrhage), cellular debris, and infiltration of moderate numbers of viable and degenerated neutrophils and increased numbers of alveolar macrophages. Around the bronchioles in the perivascular space, there was a mild to moderate infiltration of macrophages, lymphocytes, and a few plasma cells and edema. In two ferrets, there was antigen present in a few mononuclear cells of the tracheobronchial lymph node. In the other collected tissues, virus antigen expression was not demonstrated, and significant lesions were absent.

### A/H5N6 GZ/14 is not recognized by ferret antisera raised against available candidate vaccine viruses.

To study the antigenic relationship between A/H5N6 GZ/14 and other clade 2.3.4.4 viruses and the neutralization of A/H5N6 GZ/14 by postinfection antisera raised against available candidate vaccine viruses (CVVs), we performed a hemagglutination inhibition (HI) assay (Table 4). Apart from the homologous antisera, A/H5N6 GZ/14 was recognized only by antisera raised against clade 2.3.4.4 A/H5N2 A/goose/Eastern China/1112/2011 (gs/EC/11). Therefore, none of the CVVs elicited HI antibodies that cross-reacted with the A/H5N6 GZ/14 HA. Postinfection antisera raised against A/H5N6 GZ/14 reacted only poorly to A/H5N8 ck/NL/14 and A/H5N2-gs/EC/11, demonstrating antigenic divergence of A/H5 viruses within clade 2.3.4.4.

**TABLE 4:** HI assay with ferret antisera raised against a panel of candidate A/H5 vaccine viruses for pandemic preparedness selected by the WHO network

Virus^a^	Subtype	Clade	HI titer^b^ against sera raised against the following virus:									
			ml/SW/02	HK/97	VN/04	IN/05	tk/TK/05	AN/05	ck/NL/14	gy/WAS/14	gs/EC/11	GZ/14
ml/SW/02	H5N9	Classic	160	480	160	30	120	80	20	<10	<10	<10
HK/97	H5N1	0	<10	**480**	320	<10	<10	<10	<10	<10	<10	<10
VN/04*	H5N1	1	<10	10	**960**	<10	<10	<10	<10	<10	<10	<10
IN/05*	H5N1	2.1	<10	30	40	**640**	<10	40	<10	<10	<10	<10
tk/TK/05*	H5N1	2.2	<10	80	80	160	**1,280**	<10	<10	<10	<10	<10
AN/05*	H5N1	2.3.4	<10	160	240	320	80	**1,280**	<10	<10	<10	<10
ck/NL/14	H5N8	2.3.4.4	<10	<10	40	<10	<10	<10	**960**	640	320	10
gy/WAS/14*	H5N8	2.3.4.4	<10	<10	<10	<10	<10	<10	240	**160**	60	<10
gs/EC/11	H5N2	2.3.4.4	<10	<10	<10	<10	<10	<10	<10	10	**160**	80
GZ/14	H5N6	2.3.4.4	<10	<10	<10	<10	<10	<10	<10	<10	160	**320**

a Viruses marked with an asterisk are A/H5 candidate vaccine viruses.

b Homologous titers are boldface and underlined.

## DISCUSSION

Here, we report on a detailed *in vitro* and *in vivo* characterization of the clade 2.3.4.4 A/H5N6 GZ/14 virus in order to evaluate the risk for humans. Sequence analysis of A/H5N6 GZ/14 virus revealed a few amino acid substitutions that have previously been described as mammalian adaptation markers for other A/H5 viruses of the GsGd lineage: 94N (25), 133A (25), and 235P (26) in HA, which have been associated with increased binding of A/H5N1 viruses to human-type receptors, and E627K in PB2, which has been associated with increased replication of influenza viruses *in vitro* and *in vivo* at temperatures equivalent to those of the mammalian upper respiratory tract (URT) (21, 22, 27). Despite the fact that our knowledge of the genetic markers of mammalian adaptation is growing, performing risk assessment using genetic data alone might be misleading because of context dependency. Therefore, we sought to perform a phenotypic characterization of A/H5N6 GZ/14 by focusing on the phenotypes that have been associated with airborne transmission of A/H5N1 GsGd viruses (28, 29): a change in receptor binding preference of HA from avian-type to human-type receptors, α2,3-SA and α2,6-SA, respectively, increased HA thermostability and acid stability and increased polymerase activity.

Using a resialylated TRBC assay, A/H5N6 GZ/14, as well as A/H5N8 ck/NL/14, was shown to display a typical pattern of avian influenza viruses by binding exclusively to α2,3-SA. These data are in accordance with previous studies, in which A/H5N6 GZ/14 and another human A/H5N6 isolate, A/Sichuan/26221/2014, were found to bind only to α2,3-SA (30, 31). Interestingly, A/H5N6 viruses isolated from healthy ducks in China bound in a similar fashion to both α2,3-SA and α2,6-SA as well as to both upper (trachea) and lower (alveoli) respiratory epithelium (32). A/H5N6 viruses isolated from wild bird carcasses in Hong Kong also displayed a dual binding phenotype (31). The substitutions that mediated the binding of these avian A/H5N6 viruses to human-type receptors were not identified. Although most clade 2.3.4.4 viruses have retained α2,3-SA specificity, it was shown that the emergence of clade 2.3.4.4 viruses was accompanied with the capacity of these viruses to bind to fucosylated sialilosides, mediated by K218Q and S223R, substitutions that are conserved in clade 2.3.4.4 HAs (33). Of note, such modifications cannot be detected using the resialylated TRBC, in which only the nature of the bondage between the terminal sialic acid and the antepenultimate sugar is evaluated.

HA acid stability has been described as an important host range factor, associated with adaptation of avian and swine influenza viruses to the human host, airborne transmission of avian viruses, and pandemic potential (28, 29, 34–37). The HAs of A/H5N6 GZ/14 and of A/H5N8 ck/NL/14 were unstable compared to those of a human A/H3N2 virus and an avian-origin A/H5N1 transmissible virus assessed in syncytium formation and thermostability assays. A/H5N6 GZ/14 and A/H5N8 ck/NL/14 do not possess known substitutions that have been reported to increase the acid stability and/or thermostability of A/H5 viruses from other clades of the GsGd lineage, such as H103Y, T315I, or K58I (HA2 numbering) (28, 29, 38, 39).

Using a minireplicon assay, we demonstrated that A/H5N6 GZ/14 possessed a high polymerase activity, which was mediated by E627K in PB2. Out of the 16 A/H5N6 human isolates, seven isolates carry the PB2-E627K, and one isolate, A/Sichuan/26221/2014, carries the PB2-D701N substitution, which has also been associated with increased replication and transmission in mammalian hosts (20, 40, 41). Acquisition of PB2-E627K might be the result of adaptation to the human host, although this substitution has also been detected in A/H5 avian virus isolates from other clades, as well as in other avian-origin influenza viruses such as A/H7N9 (42).

Of the three virus traits that conferred airborne transmissibility to A/H5N1 viruses between ferrets, A/H5N6 GZ/14 possesses only one of these traits, which is high polymerase activity. On the basis of the results of these *in vitro* assays, we predicted that A/H5N6 GZ/14 would not be transmissible via the airborne route, which was confirmed using the ferret model. Ferrets are used as animal models to study airborne transmission of influenza viruses, as it was shown that human influenza viruses transmit via the airborne route between ferrets, while avian influenza viruses do not, corresponding to what is also observed in humans (28). Our results are in accordance with a study on the transmissibility of clade 2.3.4.4 A/H5N6 viruses, isolated from healthy ducks, that were transmitted only via the direct contact route but not via the airborne route between ferrets (32). Other viruses of clade 2.3.4.4, A/H5N8 or A/H5N2, were also found to not transmit via the airborne route between ferrets (14–16).

Previous studies have reported on the low pathogenicity of reassortant viruses of clade 2.3.4.4, such as the Korean, European, and American A/H5N8 viruses and American A/H5N2 viruses in ferrets (14–16). However, in contrast to these viruses that had acquired internal genes from LPAI viruses from wild birds, A/H5N6 GZ/14 possesses internal genes from the A/H5N1 GsGd lineage and also the adaptive substitution E627K in PB2 that might increase its pathogenicity in mammalian hosts. Ferrets are used as a model organism to study the pathogenesis of influenza viruses, as their susceptibility to infection with human and avian influenza viruses is similar to that of humans, and they develop respiratory disease similar to that observed in humans. Using the ferret model, we showed that the pathogenicity of A/H5N6 GZ/14 largely depends on the route of inoculation, as described previously for A/H5N1 viruses (24). Upon intranasal inoculation, the pathogenicity of A/H5N6 GZ/14 was intermediate between the pathogenicities of A/H5N1 IN/05 and A/H5N8 ck/NL/14 (14). The virus was detected in the olfactory bulb as early as 3 dpi, and it is possible that A/H5N6 GZ/14 would also be able to spread to the brain as seen with A/H5N1 IN/05, 7 days after intranasal inoculation (24, 43). Ferrets lost on average 13% of their body weight over the time of the experiment, displayed more clinical signs than A/H5N8 ck/NL/14-inoculated ferrets, but did not progress to pneumonia as seen in A/H5N1 IN/05-inoculated animals. Differences between A/H5N6 and A/H5N1 might just be due to the route of inoculation—intranasal—which results in virus deposition in the lower respiratory tracts of some ferrets but not necessarily all (23, 24). Therefore, to assess the ability of A/H5N6 GZ/14 to cause pneumonia, which is observed in most humans infected with A/H5 GsGd viruses, ferrets were inoculated intratracheally with 10^6^ TCID_50_ of virus. Upon intratracheal challenge, all ferrets developed a severe and fatal pneumonia characterized by severe DAD. High levels of replication of A/H5N6 GZ/14 in the lungs of infected ferrets upon deposition of the virus in the lungs correlates with the study by Hui et al., in which A/H5N6 GZ/14 was shown to replicate to very high titers in lung explants (31). Sun et al. (32) also showed that A/H5N6 viruses isolated from healthy ducks were not as pathogenic in ferrets as a 2.3.4 A/H5N1 virus, but these A/H5N6 viruses carried internal genes from different origin.

To conclude, the results from this study demonstrate that an A/H5N6 virus of the G1 genotype is not fully adapted to mammalian hosts and therefore presents a low risk to humans, although it caused more severe disease in ferrets than other clade 2.3.4.4 viruses. However, considering the diversity of genotypes among A/H5N6 viruses, more extensive characterization and risk assessment of A/H5N6 viruses of different genotypes is required in order to completely assess the risks. Moreover, owing to its pathogenicity in ferrets and its antigenic divergence from other 2.3.4.4 A/H5 Gs/Gd viruses, A/H5N6 GZ/14 virus could be used as a challenge candidate for vaccination/challenge studies to assess the ability of different vaccination strategies to elicit protective responses to emerging Gs/Gd HPAI viruses.

## MATERIALS AND METHODS

### Cells.

Madin-Darby canine kidney (MDCK) cells (ATCC) were cultured in Eagle’s minimal essential medium (EMEM) (Lonza) supplemented with 10% fetal bovine serum (FBS) (Greiner), 100 U/ml penicillin (PEN) (Lonza), 100 U/ml streptomycin (STR) (Lonza), 2 mM l-glutamine (l-Glu) (Lonza), 1.5 mg/ml sodium bicarbonate (NaHCO_3_) (Lonza), 10 mM HEPES (Lonza) and 1× nonessential amino acids (NEAA) (Lonza). 293T cells were cultured in Dulbecco modified Eagle’s medium (DMEM) (Lonza) supplemented with 10% FBS, 100 U/ml PEN, 100 U/ml STR, 2 mM l-Glu, 1 mM NaHCO_3_, and 1× NEAA. Vero cells were cultured in Iscove’s modified Dulbecco’s medium (IMDM) (Lonza) supplemented with 10% FBS, 100 U/ml PEN, 100 mg/ml STR, and 2 mM l-Glu.

### Viruses.

All eight gene segments of a human virus isolate, A/H5N6 A/Guangzhou/39715/2014 (GZ/14), were amplified from copy DNA by PCR using specific primers and cloned in a modified version of the bidirectional reverse genetics plasmid pHW2000 (44). Recombinant viruses were rescued by reverse genetics upon transfection of 293T cells as previously described (44). For *in vivo* experiments, a full recombinant A/H5N6 GZ/14 virus was used. For *in vitro* experiments (thermostability, resialylated turkey red blood cell [TRBC], and hemagglutination inhibition assays), recombinant viruses containing six gene segments of A/Puerto Rico/8/1934 (PR/34), the HA without the multibasic cleavage site (MBCS) and the NA of A/H5N6 GZ/14 or A/H5N8 A/chicken/Netherlands/EMC-3/2014 (ck/NL/14) were used. Control viruses for thermostability and resialylated red blood cell assays were recombinant viruses containing seven gene segments of PR/34 and the corresponding HA without the MBCS (if applicable). Prototypic viruses used in hemagglutination inhibition assay were recombinant viruses containing six gene segments of PR/34, the HA without the MBCS, and the NA of the corresponding virus with the exception of the A/goose/Eastern China/1112/2011 and A/gyrfalcon/Washington/41088-6/2014, which was a recombinant virus carrying seven genes of PR/34 and the corresponding HA. Virus stocks were propagated up to three times in MDCK cells and titrated in MDCK cells as described below. The sequences of the virus stocks used for every experiment were checked using Sanger sequencing. Virus names were abbreviated as follows: A/Indonesia/5/2005 (IN/05), A/Netherlands/213/2003 (NL/03), A/Mallard/Sweden/49/2002 (ml/SW/02), A/Hong Kong/156/1997 (HK/97), A/Vietnam/1194/2004 (VN/04), A/turkey/Turkey/1/2005 (tk/TK/05), A/Anhui/1/2005 (AN/05), A/gyrfalcon/Washington/41088-6/2014 (gy/WAS/14), and A/goose/Eastern China/1112/2011 (gs/EC/11).

### Titrations.

MDCK cells were inoculated with 10-fold serial dilutions of virus stocks, nose swabs, throat swabs, or homogenized tissue samples. The cells were washed with phosphate-buffered saline (PBS) 1 h after inoculation and cultured in infection medium, consisting of EMEM supplemented with 100 U/ml PEN, 100 U/ml STR, 2 mM l-Glu, 1.5 mg/ml NaHCO_3_, 10 mM HEPES, 1× NEAA, and 20 μg/ml trypsin (*N*-tosyl-l-phenylalanine chloromethyl ketone [TPCK]-treated trypsin; Sigma). Three days after inoculation, supernatants of cell cultures were tested for agglutinating activity using TRBCs as an indicator of virus replication. Infectious virus titers were calculated from 4 replicates each of the homogenized tissue samples, nose swabs, and throat swabs and from 10 replicates of the virus stocks by the method of Reed and Muench (45).

### Ferret experiments.

Ferrets were housed and experiments were conducted in strict compliance with European guidelines (European Union [EU] directive on animal testing 86/609/EEC) and Dutch legislation (Experiments on Animals Act, 1997). All animal experiments were approved by the independent animal experimentation ethical review committee “stichting dier experimenten commissie (DEC) consult” (Erasmus MC permit number EMC4028). Research projects involving laboratory animals can be executed only if they are approved by the animal experiments committee (DEC). The DEC considers the application and pays careful attention to the effects of the intervention on the animal and its discomfort and weighs this against the social and scientific benefit to humans or animals. The researcher is required to keep the effects of the intervention to a minimum, based on the three R’s (refinement, replacement, reduction).

### (i) Ferret transmission experiments.

Four ferrets were inoculated intranasally with a total dose of 10^6^ TCID_50_ of virus distributed into the two nostrils (250 µl per nostril). Each donor ferret was then placed in a transmission cage. One day after inoculation, one naive recipient ferret was placed opposite of each donor ferret. Each transmission pair was housed in a separate transmission cage designed to prevent direct contact but to allow airflow from the donor ferret to the recipient ferret. Nose and throat swabs were collected at 1, 3, 5, and 7 days postinoculation (dpi) from donor ferrets and at 1, 3, 5, 7, and 9 days postexposure (dpe) from the recipient ferrets and subjected to endpoint titration in MDCK cells. Donor ferrets were euthanized at 7 dpi and recipient ferrets were euthanized at 14 dpe to allow assessment of seroconversion.

### (ii) Ferret pathogenesis experiments.

Six ferrets were inoculated intranasally with a total dose of 10^6^ TCID_50_ of A/H5N6 GZ/14 by applying 250 μl of virus suspension dropwise to each nostril. Clinical scores in all groups were assessed every day. Activity status was scored as follows: 0, alert and playful; 1, alert and playful only when stimulated; 2, alert but not playful when stimulated; 3, neither alert nor playful when stimulated. Dyspnea was characterized by open-mouth breathing with exaggerated abdominal movement (46). Body weight was recorded daily. Throat and nose swabs were collected every day and were stored at −80°C in transport medium (Hanks’ balanced salt solution containing 0.5% lactalbumin [Sigma], 10% glycerol [Sigma], 200 U/ml PEN, 200 mg/ml STR, 100 U/ml polymyxin B sulfate [Sigma], and 250 mg/ml gentamicin [Gibco]) for endpoint titration in MDCK cells. At 3 and 6 dpi, three animals from each group were euthanized by exsanguination under anesthesia and were necropsied according to a standard protocol (46). Tissues harvested for virological examination (right nasal turbinates, trachea, right bronchus, right lungs, tracheobronchial lymph nodes, right tonsils, right olfactory bulb, right cerebrum, right cerebellum, heart, liver, spleen, kidney, adrenal gland, pancreas, and jejunum) were homogenized in transport medium using the FastPrep system (MP Biomedicals) with two one-quarter-inch ceramic sphere balls, centrifuged 1,500 × *g* for 10 min, aliquoted, and stored at −80°C for endpoint titration in MDCK cells. Tissues harvested for histological examination (nasal turbinates, larynx, trachea, left bronchus, left lung, tracheobronchial lymph nodes, heart, liver, spleen, kidney, adrenal gland, esophagus, duodenum, jejunum, colon, pancreas, mesenteric lymph node, left olfactory bulb, left cerebrum, left cerebellum, and eye lid) were fixed in 10% neutral buffered formalin, embedded in paraffin, sectioned at 4 μm, and stained with hematoxylin and eosin (HE) for examination by light microscopy.

In a second experiment, three ferrets were inoculated intratracheally with 3 ml of virus dilution containing 10^6^ TCID_50_. The experimental design and procedures were similar to those performed for the intranasal inoculation.

### Immunohistochemistry (IHC).

For detection of influenza A virus antigen, sequential slides of all tissues were stained with a primary antibody against the influenza A virus nucleoprotein (NP) as described previously (46). In each staining procedure, an isotype control was included as a negative control, and a lung section from a cat infected experimentally with A/H5N1 was used as a positive control.

### Resialylated turkey red blood cell assay.

All sialic acids (SA) were removed from the surfaces of TRBCs by incubating 1% TRBCs in PBS with 50 mU of *Vibrio cholerae* NA (VCNA) (Roche) in 8 mM calcium chloride (CaCl_2_) at 37°C for 1 h. After two washing steps with PBS, VCNA-treated TRBCs were resuspended in PBS containing 1% bovine serum albumin (BSA) to a final concentration of 0.5% TRBCs. Removal of SA was confirmed by observation of a complete loss of hemagglutination of the TRBCs by control influenza A viruses. Subsequently, resialylation was performed using 0.5 mU of α2,3-*N*-sialyltransferase (Sigma) or 25 mU of α2,6-*N*-sialyltransferase (Sigma) and 1.5 mM CMP-sialic acid (Merck) at 37°C for 2 h in a final volume of 75 μl to produce α2,3-TRBCs and α2,6-TRBCs, respectively. After two washing steps, the TRBCs were resuspended in PBS containing 1% BSA to a final concentration of 0.5% TRBCs. Resialylation was confirmed by hemagglutination of viruses with known receptor specificity: A/H5N1 IN/05 and A/H3N2 NL/13. The receptor specificity of A/H5N8 ck/NL/14 and A/H5N6 GZ/14 was tested by performing a standard hemagglutination assay with the modified TRBCs.

### Syncytium formation assay.

Influenza virus HA-induced cell fusion was tested in Vero-118 cells transfected with 5 μg of HA cloned in pCAGGs expression plasmid using Xtremegene transfection reagent according to the manufacturer’s instructions (Roche). One day after transfection, the cells were harvested using trypsin-EDTA and plated in 24-well plates. The next morning, the cells were washed, and medium was replaced with IMDM (Lonza) containing 10 μg/ml of trypsin. After 1 h, the cells were washed with PBS and exposed to PBS at pH 4.8, 4.9, 5.0, 5.1, 5.2, 5.3, 5.4, 5.5, 5.6, 5.7, 5.8, or 5.9 for 10 min at 37°C. Subsequently, the PBS was replaced by IMDM supplemented with 10% FBS, 100 U/ml PEN, 100 mg/ml STR, and 2 mM l-Glu. The next day, the cells were fixed using 80% ice-cold acetone, washed, and stained using a 20% Giemsa solution (Merck Millipore, Darmstadt, Germany). Visual inspection of the cell cultures for the presence of syncytia (multinucleated cells) was used to determine the pH threshold required for fusion.

### Thermostability assay.

In short, viruses were diluted to 128 HA units (HAU)/25 μl using PBS. The samples were incubated in a thermal cycler for 30 min at temperatures of 50°C, 51.7°C, 54.3°C, 56°C, 58.5°C, and 60°C. Subsequently, the HA titer was determined by performing a hemagglutination assay using TRBCs.

### Polymerase assay.

293T cell monolayers were transfected with 125 ng of luciferase reporter plasmid (firefly open reading frame flanked by the noncoding regions of the M segment under control of a PolI promoter) and 12.5 ng of an internal control plasmid (*Renilla* gene under the cytomegalovirus [CMV] promoter) together with the mix of PB2, PB1, PA, and NP plasmids, cloned in a bidirectional reverse genetics plasmid, in quantities of 125, 125, 125, and 250 ng, respectively. The transfected cells were incubated at 33°C or 37°C. After 24-h incubation, the supernatants were discarded, and the cell extracts were prepared in 100 μl of lysis buffer. The luciferase levels were assayed with luciferase assay system (Promega) and detected by a luminometer. Polymerase complexes of control viruses were included in the assay: a human H1N1 virus A/Hong Kong/54/1998 (HK/98), two human A/H5N1 viruses A/Vietnam/1203/2004 (VN/1203/04) and A/Indonesia/5/05 (IN/05), and an avian virus A/H5N2 A/mallard/Netherlands/3/1999 (ml/NL/99).

### Hemagglutination inhibition assay.

Ferret antisera were prepared by intranasal inoculation with 500 µl recombinant virus containing six gene segments of PR/34 (47), the corresponding HA without the MBCS, and the corresponding NA. Fourteen days postinoculation, ferrets were boosted by injecting subcutaneously 500 µl of a 1:1 mix of concentrated virus (>2,048 HAU) and Titermax Gold adjuvant (Sigma-Aldrich, St. Louis, MO, USA). Antisera were collected 2 weeks later. Ferret antisera were then pretreated overnight with receptor-destroying enzyme (VCNA) at 37°C and incubated at 56°C for 1 h the next day. Antisera were subsequently treated with 10% TRBCs at 4°C for an hour. Twofold serial dilutions of the antisera, starting at a 1:20 dilution, were mixed with 25 μl of a virus stock containing 4 hemagglutinating units and were incubated at 37°C for 30 min. Subsequently, 25 μl of 1% TRBCs was added, and the mixture was incubated at 4°C for 1 h. Hemagglutination inhibition (HI) was read and was expressed as the reciprocal value of the highest dilution of the serum that completely inhibited agglutination of virus and erythrocytes. The detection limit corresponds to half of the first antisera dilution (<10).

### Biosafety.

All experiments were conducted within the enhanced animal biosafety level 3 (ABSL3+) facility of Erasmus MC. The ABSL3+ facility consists of a negative-pressure laboratory (−30 Pa) in which all *in vivo* and *in vitro* experimental work is carried out in class 3 isolators or class 3 biosafety cabinets, which also have negative pressure (less than −200 Pa). Although all experiments are conducted in closed class 3 cabinets and isolators, special personal protective equipment, including laboratory suits, gloves, and FFP3 facemasks, is used. Air released from the class 3 units is filtered by high-efficiency particulate air (HEPA) filters and then leaves via the facility ventilation system, again via HEPA filters. Only authorized personnel that have received the appropriate training can access the ABSL3+ facility. For animal handling in the facilities, personnel always work in pairs. The facility is secured by procedures recognized as appropriate by the institutional biosafety officers and facility management at Erasmus MC and Dutch and U.S. government inspectors.
